# Quantitative Profiling of Serum Carnitines Facilitates the Etiology Diagnosis and Prognosis Prediction in Heart Failure

**DOI:** 10.3390/molecules28145345

**Published:** 2023-07-11

**Authors:** Zhangwei Chen, Danbo Lu, Baoling Qi, Yuan Wu, Yan Xia, Ao Chen, Su Li, Huiru Tang, Juying Qian, Junbo Ge

**Affiliations:** 1 Department of Cardiology, Zhongshan Hospital, Fudan University, 180 Fenglin Road, Xuhui District, Shanghai 200032, China; chen.zhangwei@zs-hospital.sh.cn (Z.C.); lu.danbo@zs-hospital.sh.cn (D.L.); watson_woo@163.com (Y.W.); xia.yan@zs-hospital.sh.cn (Y.X.); chen.ao@zs-hospital.sh.cn (A.C.); li.su@zs-hospital.sh.cn (S.L.); 2 Shanghai Institute of Cardiovascular Diseases, 180 Fenglin Road, Xuhui District, Shanghai 200032, China; 3 National Clinical Research Center for Interventional Medicine, 180 Fenglin Road, Xuhui District, Shanghai 200032, China; 4 State Key Laboratory of Genetic Engineering, Zhongshan Hospital and School of Life Sciences, 2005 Songhu Road, Yangpu District, Shanghai 200438, China; qbl@smi-wh.cn; 5 Human Phenome Institute, Metabonomics and Systems Biology Laboratory at Shanghai International Centre for Molecular Phenomics, Fudan University, 2005 Songhu Road, Yangpu District, Shanghai 200438, China; 6 Shanghai Metabolome Institute-Wuhan (SMI), No.128 Guanggu 7 Road, East Lake High-Tech Development Zone, Wuhan 430074, China

**Keywords:** heart failure, carnitines, myocardial metabolism, ischemic heart disease, dilated cardiomyopathy

## Abstract

Background: The perturbation of fatty acid metabolism in heart failure (HF) has been a critical issue. It is unclear whether the amounts of circulating carnitines will benefit primary etiology diagnosis and prognostic prediction in HF. This study was designed to assess the diagnostic and prognostic values of serum carnitine profiles between ischemic and non-ischemic derived heart failure. Methods: HF patients (non-ischemic dilated cardiomyopathy: DCM-HF, *n* = 98; ischemic heart disease: IHD-HF, *n* = 63) and control individuals (*n* = 48) were enrolled consecutively. The serum carnitines were quantitatively measured using the UHPLC-MS/MS method. All patients underwent a median follow-up of 28.3 months. Multivariate Cox regression analysis was performed during the prognosis evaluation. Results: Amongst 25 carnitines measured, all of them were increased in HF patients, and 20 acylcarnitines were associated with HF diagnosis independently. Seven acylcarnitines were confirmed to increase the probability of DCM diagnosis independently. The addition of isobutyryl-L-carnitine and stearoyl-L-carnitine to conventional clinical factors significantly improved the area under the receiver operating characteristic curve (ROC) from 0.771 to 0.832 (*p* = 0.023) for DCM-HF diagnosis (calibration test for the composite model: Hosmer-Lemeshow χ^2^ = 7.376, *p* = 0.497 > 0.05). Using a multivariate COX survival analysis adjusted with clinical factors simultaneously, oleoyl L-carnitine >300 nmol/L (HR = 2.364, 95% CI = 1.122–4.976, *p* = 0.024) and isovaleryl-L-carnitine <100 nmol/L (HR = 2.108, 95% CI = 1.091–4.074, *p* = 0.026) increased the prediction of all-cause mortality independently, while linoleoyl-L-carnitine >420 nmol/L, succinyl carnitine >60 nmol/L and isovaleryl-L-carnitine <100 nmol/L increased the risk of HF rehospitalization independently. Conclusions: Serum carnitines could not only serve as diagnostic and predictive biomarkers in HF but also benefit the identification of HF primary etiology and prognosis.

## 1. Introduction

Heart failure remains to be a major cardiac disease and public health burden worldwide, even though a series of therapeutic techniques and strategies have been established. Novel explorations of HF pathophysiological mechanism, such as angiotensin up-regulation; unfavorable increase of sympathetic excitability increasing; and aldosterone mediated sodium retention initiated the anti-neurohormonal era of HF optimal management [1]. However, current practicable predictors, including left ventricular ejection fraction (LVEF), natriuretic peptide levels and New York Heart Association (NYHA) class, could not fully explain and predict the risk of long-term adverse outcomes in chronic HF patients.

Novel metabolic profiling with metabolomic technologies in clinical researches has shown promising potentials as diagnostic/predictive tools and for optimizing the management of cardiovascular disease [2,3,4]. It is well known that a metabolism dysfunction of fatty acids in the myocardium occurs gradually in heart failure progression [5,6,7]. The turbulence of L-carnitine, which transports fatty acids across the mitochondrial membrane for β-oxidation, plays a critical role on cardiac remodeling and HF progression. Carnitine deficiency may cause myocardial dysfunction and heart failure, and L-carnitine supplementation results in a beneficial effect for heart failure patients [8]. Short-chain acyl-carnitines generated from branch chains amino acids (including valine, leucine and isoleucine) are also indicators for mitochondrial functions (or dysfunctions) [9,10]. This indicates that quantitative profiling of serum carnitines may provide a unique opportunity to obtain catabolic information of both fatty acids and amino acids associated with HF progression. Furthermore, the common etiologies of HF can be categorized into ischemic heart disease (IHD) and non-ischemic dilated cardiomyopathy (DCM) in clinical settings, distinguished by the extent of coronary artery stenosis mostly. These two crucial etiologies have distinct long-term clinical prognoses and need different strategies of treatment [11,12]. However, few studies were performed to differentiate the detailed serum carnitine profiles between IHD-HF and DCM-HF patients.

Here, therefore, we designed this clinical observation study to (1) define serum carnitines profiles in HF patients and their diagnostic value for HF, (2) evaluate the efficiency of multiple carnitines for ischemic and non-ischemic HF differential diagnosis and (3) clarify the impact of serum carnitines on long-term cardiac events.

## 2. Results

### 2.1. Clinical Baseline Characteristics of Enrolled Patients in This Study

**Figure 1:**
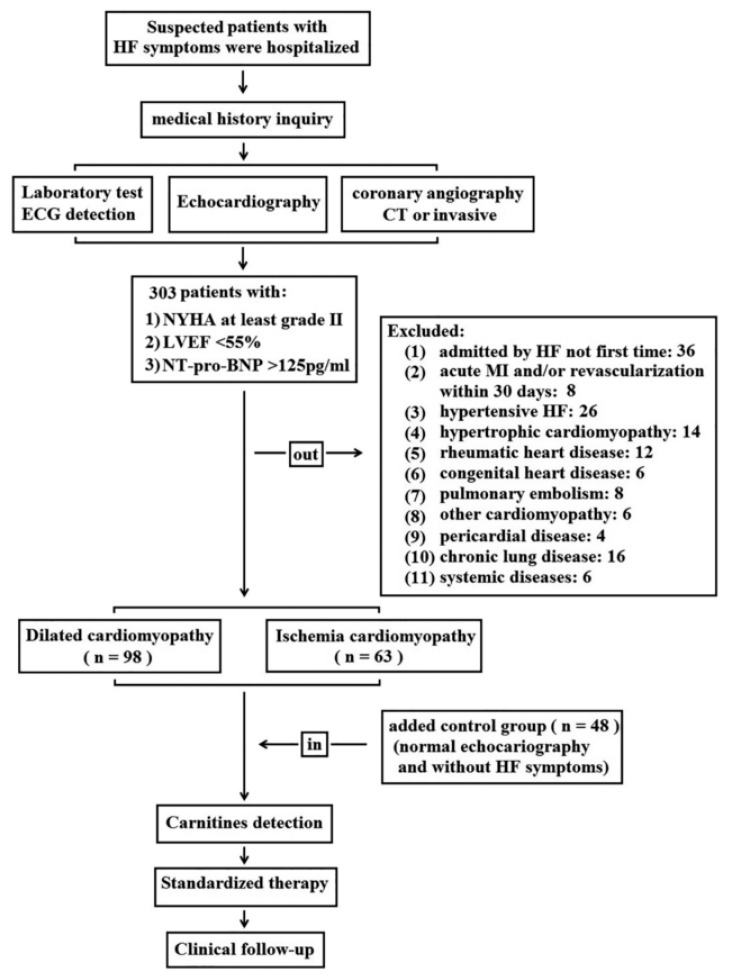
Study flowchart (HF: heart failure; ECG: electrocardiography; CT: computerized tomography; NYHA: New York Heart Association; LVEF: left ventricular ejection fraction; MI: myocardial infarction).

From February to September in 2017, a total of 161 HF patients (DCM-HF: *n* = 98; IHD-HF: *n* = 63) and 48 control patients were enrolled into our study consecutively (Figure 1: study flow chart). The mean age of patients at the time of enrollment was 62 years in the HF group and 47 years in the control group. The baseline characteristics were shown in Table 1.

Heart functions in both the DCM-HF and IHD-HF groups were much worse than in the control group. We could also find that the DCM-HF patients had much lower LVEF (32.8 ± 7.9% vs. 37.9 ± 7.8%, *p* < 0.01) and higher left atrial diameter (51.6 ± 10.0 mm vs. 47.1 ± 7.6 mm, *p* < 0.01) than IHD-HF patients.

### 2.2. Levels of Serum Carnitines and Other Traditional Biomarkers at Baseline

A total of 27 different carnitines were detected with 25 acyl-carnitines being increased significantly in HF groups (Table 2). Carnitine (C0) itself was only increased slightly in IHD-HF (5.12%) and DCM-HF (14.11%) groups. NT-pro-BNP, Troponin T and hs-CRP, as traditional biomarkers, were also increased significantly in HF group.

### 2.3. Multivariate Logistic Analysis of the Impact of Carnitines on HF Diagnosis

ROC was analyzed to identify HF diagnosis. The AUC to HF diagnosis showed significant values for baseline C4DC (AUC = 0.915, *p* < 0.01), C20:4 (AUC = 0.871, *p* < 0.01), C18:1 (AUC = 0.870, *p* < 0.01) and C18:2 (AUC = 0.868, *p* < 0.01), shown in Table 3.

In the multivariate logistic analysis for HF diagnosis, we found age, male gender, hypertension and serum creatinine increased HF diagnosis independently among clinical risk factors. Then, each carnitine was evaluated for its independent impact on HF diagnosis, adjusted simultaneously by age, creatinine, male gender and hypertension. It was also demonstrated that several carnitines verified a HF diagnosis independently, shown in Table 3.

### 2.4. Baseline Carnitines Discriminated DCM-HF from IHD-HF

**Figure 2:**
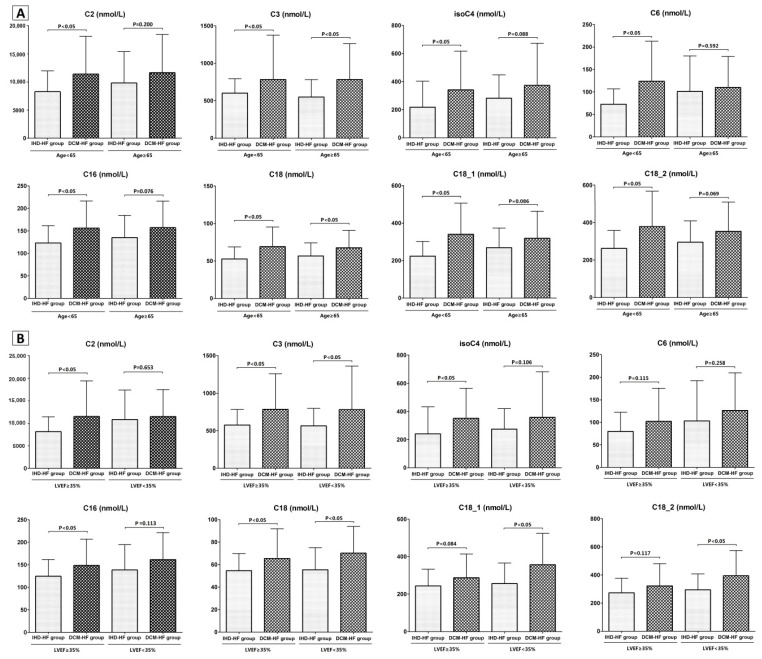
Carnitines in different age (**A**) and LVEF (**B**) subgroup-stratification analyses.

More interestingly, the levels of several acyl-carnitines were quite different between IHD-HF and DCM-HF groups (Table 2), even in age and LVEF subgroup-stratification analyses (Figure 2).

The AUC to predict DCM-HF showed significant values for baseline acyl-carnitines. Furthermore, even adjusted by age, LDL-C, serum uric acid and LVEF simultaneously, seven acyl-carnitines (C2, C3, isoC4, C6, C18, C18:1 and C18:2) still increased the DCM-HF discrimination independently, shown in Table 4.

### 2.5. Carnitines Added to Clinical Factors for DCM-HF Discrimination

**Figure 3:**
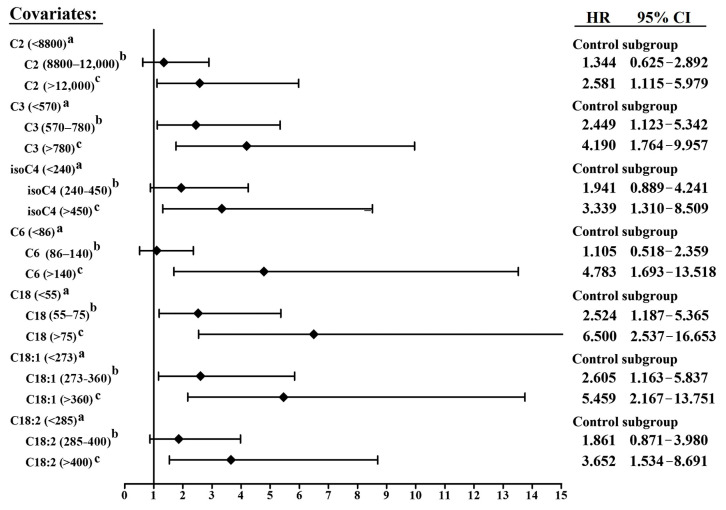
Forest plots demonstrated the impact of carnitines on DCM-HF diagnosis. a: low concentration; b: medium concentration; c: high concentration.

Forest plots were applied to demonstrate the impact of different levels of carnitines on hazard ratios (HR) of the DCM-HF diagnosis, Figure 3.

According to univariate logistic analysis, clinical factors of age, LDL-C (excluded in the model establishment because of possible statin treatments in the outpatient department), serum uric acid, troponin T and LVEF were associated with higher probability of DCM-HF. Then, baseline C18, C18:2, C3, C2, C6, isoC4 (C18:1 was excluded with the test of multi-collinearity, VIP = 8.264) combined with clinical factors (age, uric acid, troponin T and LVEF) were set as independent variables in multivariate logistic analysis for DCM-HF diagnosis.

**Figure 4:**
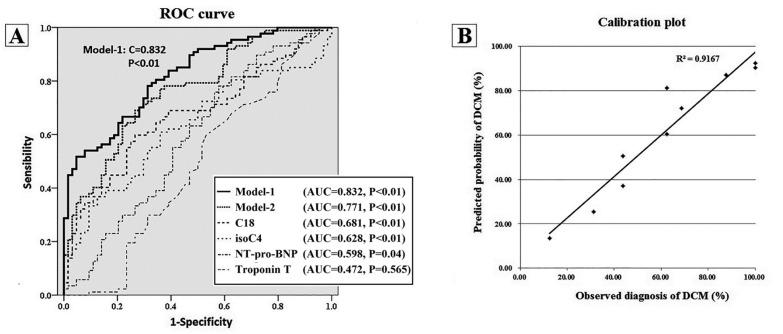
The prediction model combining carnitines and clinical factors showed good discrimination for DCM-HF. (**A**) ROC Curves of prediction models, isoC4, C18, troponin T and LVEF to discriminate DCM-HF. (**B**) Calibration plot indicated a good calibration of Model-1 in DCM-HF diagnosis. (DCM: dilated cardiomyopathy; HF: heart failure).

Finally, isoC4, C18, age, troponin T and LVEF remained in the prediction model, while we saved each probability value as a novel independent variable (Model-1). In the discrimination test, the AUC to predict DCM-HF for Model-1 reached 0.832 (*p* < 0.01), which indicated a good discrimination. Such an AUC was significantly higher (*p* = 0.023) than those from Model-2 (only enrolled clinical factors: AUC = 0.771), C18 and isoC4, shown in Figure 4A (calibration test: Hosmer–Lemeshow χ^2^ = 7.376, and *p* = 0.497 and calibration plot R^2^ = 0.9167, which indicated a good calibration of Model-1 in DCM-HF diagnosis, in Figure 4B).

### 2.6. Baseline Carnitines and Primary Clinical Outcomes

During a median follow-up of 28.3 months, 12 patients (7.5%) were lost to follow-up. A total of 43 mortality events occurred including 18 death (31.6%) in the IHD-HF group and 25 (27.2%) in the DCM-HF group (*p* = 0.564) whereas there were 54 HF rehospitalization including 14 (24.6%) in the IHD-HF group and 40 (43.5%) in the DCM-HF group (*p* = 0.020).

#### 2.6.1. All-Cause Mortality

**Figure 5:**
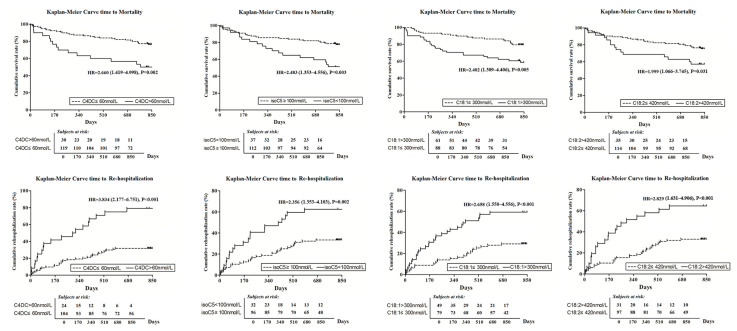
Kaplan–Meier curves showed that the change of several carnitines increased the risk of mortality and rehospitalization in heart failure patients.

Univariate COX regression analysis indicated that the risk of all-cause mortality in HF patients was increased with age > 65 (HR = 1.978, 95% CI = 1.056–3.704, *p* = 0.033), serum creatinine > 2 mg/dL (HR = 7.687, 95% CI = 3.191–18.518, *p* < 0.01), NT-pro-BNP > 4000 pg/mL (HR = 3.184, 95% CI = 1.705–5.947, *p* < 0.01) and left atrial diameter > 55 mm (HR = 2.847, 95% CI = 1.482–5.467, *p* < 0.01). However, we did not find a significant impact from LVEF < 35% (HR = 1.106, 95% CI = 0.607–2.014, *p* = 0.742) nor from the diagnosis of DCM (HR = 0.822, 95% CI = 0.449–1.507, *p* = 0.527) on mortality. Nevertheless, several acyl-carnitines showed significant increases of the mortality risk (Figure 5).

In multivariate COX regression, C18:1 >300 nmol/L (HR = 2.363, 95% CI = 1.122–4.976, *p* = 0.024) and isoC5 < 100 nmol/L (HR = 2.108, 95% CI = 1.091–4.074, *p* = 0.026) still significantly increased the risk of all-cause mortality independently after being adjusted for age, serum creatinine, NT-pro-BNP and left atrial diameter, Table 5.

#### 2.6.2. HF Rehospitalization Rate

In univariate COX regression analysis, the incidence of HF rehospitalization rate was significantly increased with age > 65 (HR = 1.793, 95% CI = 1.037–3.099, *p* = 0.037), diagnosis of DCM-HF (HR = 1.900, 95% CI = 1.033–3.495, *p* = 0.039), LVEF < 35% (HR = 3.380, 95% CI = 1.836–6.221, *p* < 0.01), left atrial diameter > 55 mm (HR = 2.410, 95% CI = 1.325–4.382, *p* < 0.01), serum creatinine > 2 mg/dL (HR = 3.572, 95% CI = 1.415–9.016, *p* < 0.01), NT-pro-BNP > 4000 pg/mL (HR = 2.659, 95% CI = 1.556–4.543, *p* < 0.01). Furthermore, a series of carnitines also significantly increased the rate of HF rehospitalization (Figure 4).

In multivariate COX regression analysis, even adjusted by these six clinical factors (age > 65, diagnosis of DCM-HF, LVEF < 35%, left atrial diameter > 55 mm, serum creatinine > 2 mg/dL and NT-pro-BNP > 4000 pg/mL) together with C18:2 > 420 nmol/L, C4DC > 60 nmol/L and isoC5 < 100 nmol/L also increase the incidence of HF rehospitalization independently, Table 5.

## 3. Methods

### 3.1. Patient Enrollment and Ethical Approval

From Jan to Sep in 2017, patients suffering symptoms and signs of chronic HF for the first time, such as chest distress, shortness of breath, dyspnea, pulmonary rales, lower extremity edema, were admitted to our department and screened further. Eligibility requirements of HF patients for enrollment included (1) age >18 years, (2) left ventricular ejection fraction (LVEF) <55%, (3) plasma N-terminal pro-B-type natriuretic peptide (NT-pro-BNP) level ≥125 pg/mL [1] and (4) provision of written informed consent for enrollment and data publication. The exclusion criteria included (1) New York Heart Association (NYHA) class I; (2) suffered from acute myocardial infarction (MI) and revascularization within 30 days; (3) not first-time admitted to hospital because of HF; (4) etiologies of HF were caused by hypertension, hypertrophic cardiomyopathy, rheumatic heart disease, severe arrhythmia, congenital heart disease or other cardiomyopathy (excepted DCM or IHD); (5) pericardial disease; (6) chronic lung disease; (7) pulmonary embolism; and (8) symptoms caused by systemic diseases including anemia, hyperthyroidism/hypothyroidism, renal failure, dysfunction of hematological and immunological, carcinoma or a condition treated with immunosuppressive agents.

A total of 161 HF patients (dilated cardiomyopathy: DCM-HF, n = 98; ischemic heart disease: IHD-HF, *n* = 63) and control patients (*n* = 48) were enrolled consecutively; the study flow chart is shown in Figure 1). Patients in the control group were admitted to hospital for routine physical examination originally with normal cardiac function (verified with echocardiography and NT-pro-BNP tests) and had no HF symptoms and signs.

The study protocol was approved by the Ethics Committee of Zhongshan Hospital (Approval NO.: B2016-019R, Date: 16 March 2016) and registered in Clinicaltrials.gov (NCT03797742). All HF patients and controls provided written informed consent. The study was conducted in accordance with the guidelines of the Declaration of Helsinki.

### 3.2. Clinical Detection and Information Collection

#### 3.2.1. Laboratory Measurements

During hospitalization, fast blood samples at admission were obtained. Serum and plasma were isolated for blood biochemistry and cardiac injury biomarker analyses. Frozen samples at −80 °C were cold-chain transported to Shanghai Metabolome Institute-Wuhan (SMI) for quantitative measurements of carnitines (See Section 3.3). Serum creatinine, uric acid, high-sensitivity C-reactive protein (hs-CRP) and N-terminal pro–B-type natriuretic peptide (NT-proBNP) were measured. Serum creatinine, uric acid and high-sensitivity C-reactive protein (hs-CRP) were measured using standard ARCHITECT immunoassays (Abbott Laboratories, Abbott Park, IL, USA). Troponin T was measured with high-sensitivity electrochemiluminescence immunoassay (Roche Diagnostics, Basle, Switzerland) with a lower limit of detection of 5 ng/L. Plasma N-terminal pro–B-type natriuretic peptide (NT-proBNP) was measured with a sandwich immunoassay (Roche Diagnostics) with a reporting range of 25–35,000 pg/mL. All assays were performed by laboratory technicians blinded to clinical diagnosis and outcomes.

#### 3.2.2. Echocardiography and Electrocardiograph

Transthoracic echocardiography and 12-lead electrocardiograph were performed within 24 h after patient admission to hospital. Observers were blinded to the results of clinical data and any laboratory tests. Left ventricular ejection fraction (LVEF) was measured together with left ventricular end-diastolic dimension (LVEDD), left ventricular end-systolic dimension (LVESD), left atrial diameter (LA) and mean pulmonary artery pressure (mPAP).

#### 3.2.3. Coronary Angiography

After the medical history was collected and the status of water–sodium retention was regulated, coronary CT angiography or invasive coronary angiography (mostly punctured by radial artery) was arranged for HF patients within 2–5 days after admission. Coronary stenoses in left main (LM), left anterior descending artery (LAD), left circumflex artery (LCX) and right coronary artery (RCA) were identified with a visual consensus of two experienced interventional physicians, who were also blinded to patients’ clinical information.

#### 3.2.4. Etiology Diagnosis of HF and Treatment Strategy

The proposed definition of ischemic heart disease (IHD) was patients with (1) history of MI or revascularization (either percutaneous coronary intervention (PCI) or coronary artery bypass grafting), (2) ≥75% stenosis of left main or proximal LAD, (3) ≥75% stenosis of two or more epicardial vessels [1,12]. The diagnostic criterion was in accordance with ESC guidelines [13].

Patient management and therapeutic strategy were at the discretion of the treating physicians and heart failure specialists and were provided in accordance with clinical guidelines [1,14]. These strategies were also optimized gradually by the alteration of clinical manifestation and detection reports.

### 3.3. Quantitative Measurements of Serum Carnitines

Carnitines in serum samples including free L-carnitine, short-chain (≤C6), medium-chain (C8-12) and long-chain (C14-22), were quantified by using an UHPLC-MS/MS method described previously. Such analysis was conducted on an Agilent system consisting of a 1290 UHPLC-system coupled with an Agilent 6470 triple-quadrupole mass spectrometer (Agilent Technologies, Palo Alto, CA, USA). For the convenience of discussion, acyl-carnitines were denoted as their correspondent acyl name. For example, acetyl-carnitine, succinyl-carnitine and palmitoyl-carntine were denoted as C2, C4DC and C16:0, respectively.

#### 3.3.1. Reagents

AR grade methanol was purchased from SCRC (Sinopharm Chemical Reagent Co., Ltd., Shanghai, China). HPLC grade formic acid and acetonitrile were purchased from Thermo Fisher (Thermo Fisher Scientific Inc., Waltham, MA, USA). In total, 27 carnitines and 2 13C-labeled internal standards (ISs) were purchased from Sigma-Aldrich (Sigma-Aldrich, Inc., St. Louis, MI, USA), TRC (Toronto Research Chemicals, Toronto, ON, Canada) and Santa Cruz (Santa Cruz Biotechnology, Inc., Dallas, TX, USA).

#### 3.3.2. Sample Preparation

Serum samples (total volume of 20 μL) and 10 μL of internal standard (1 μM Acetyl-13C2- L-carnitine and 0.1 μM Palmitoyl-1,2,3,4-13C4-L-carnitine in 80% methanol) were subjected to protein precipitation by adding 70 μL of precooled methanol. Samples were centrifuged at 12,000× *g* rpm for 10 min at 4 °C, and the supernatants were filtered with a 0.22 µm membrane filter before UPLC-MS/MS analysis.

#### 3.3.3. Calibration Curves

Calibration curve linearity was evaluated by assessing the correlation coefficient (R2) of three freshly prepared 15-point calibration curves. Moreover, 1–35 mmol/L carnitine stock solutions were prepared by dissolving an appropriate amount of each compound in methanol. The carnitine stock solutions were then combined to form standard stock solutions and diluted with solvent of 80% methanol to a final concentration of 1000 nmol/L. A combined internal standards stock solution composed of 1000 nmol/L Acetyl-13C2-L-carnitine and 100 nmol/L Palmitoyl-1,2,3,4-13C4-L-carnitine was also prepared in 80% methanol. Calibration curves were generated by diluting the stock solutions to 1000, 500, 250, 100, 50, 25, 10, 5, 2.5. 1, 0.5, 0.25, 0.1, 0.05 and 0.025 nmol/L in 80% methanol using volumetric glassware; then 90 μL every point of standard mixture was mixed with 10 μL internal standards stock solution, and the above extraction procedure was repeated twice. Standard curves were constructed with least-squares linear regression analysis using the peak area ratio of a given carnitine over its reference IS against the nominal concentration of the calibrator.

#### 3.3.4. Liquid Chromatography and Mass Spectrometry

UPLC-MS/MS analyses were conducted on an Agilent UPLC-MS/MS system consisting of an 1290 UPLC-system coupled with an Agilent 6470 triple-quadrupole mass spectrometer (Agilent Technologies, USA) [15,16,17]. For analysis, 1 μL of the extraction was injected. Chromatographic separation was achieved on an Agilent ZORBAX RRHD Eclipse XDB C18 column (2.1 × 100 mm, 1.8 µm particles) using a flow rate of 0.5 mL/min at 40 °C during a 10 min gradient (0–1 min 1% B, 1–3 min from 1% B to 15% B, 3–5 min from 15% B to 65% B, 5–7 min from 65% B to 95% B, 7–10 min 95%, then followed by 3 min post-run for column re-equilibration), while using the solvents A, water containing 0.1% formic acid, and B, acetonitrile containing 0.1% formic acid. Electrospray ionization was performed in the positive ion mode using N2 at a pressure of 50 psi for the nebulizer with a flow of 10 L/min and a gas temperature of 315 °C, respectively. The sheath gas temperature was 350 °C with a flow rate of 10 L/min. The capillary was set at 4000 V. Multiple reactions monitoring (MRM) has been used for quantification of screening fragment ions.

#### 3.3.5. Data Preprocessing

Peak determination and peak area integration were performed with MassHunter Workstation software (Agilent, Version B.08.00, Palo Alto, CA, USA) while auto-integration was manually inspected and corrected if necessary. The obtained peak areas of targets were corrected with appropriate internal standards (IS), and calculated response ratios were used throughout the analysis.

#### 3.3.6. Quantitation of Carnitines without Commercial Standards

**Figure 6:**
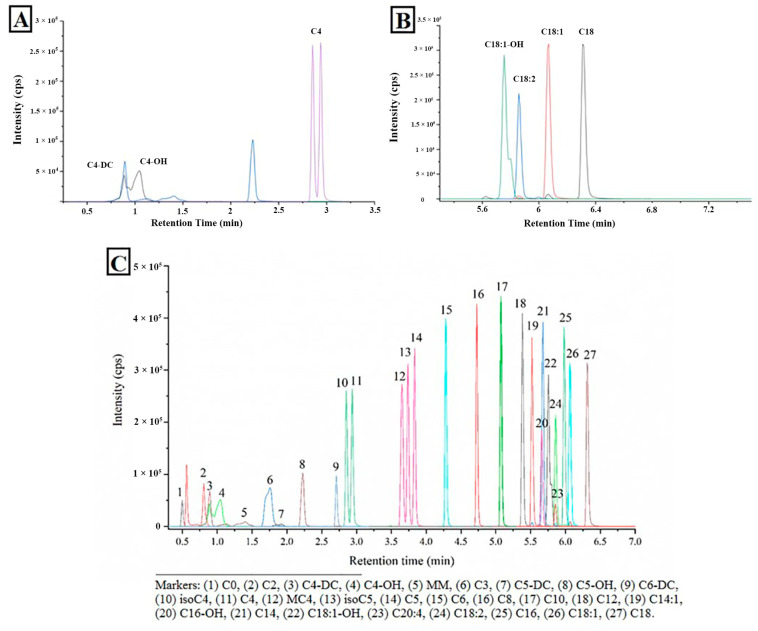
Retention pattern of Carnitines ((**A**): C4; (**B**): C18; (**C**): All).

Commercially available standards of carnitines are limited. Therefore, the quantification of those carnitines for which direct standards could not be obtained is complicated. Identification of these was based on the measured mass, fragmentation pattern and chromatographic properties. The product ion of *m*/*z* 85.1 was used as a quantification ion, and the product ion of *m*/*z* 60.2 as a quantitative ion. Carnitines with the same carbon numbers but different substituent group show some regularities; for example, C4DC elute fastest on column, and then C4OH, C4 elute slowest (Figure 6). The concentrations of those carnitines were calculated based on the calibration curve of a suitable standard carnitine which was in close proximity on the chemical construction.

### 3.4. Follow-Up and Primary Outcomes

All HF patients underwent clinical followed-up via an outpatient clinic attendance or a telephone consultation after hospital discharge. Information on each outpatient visit or telephone consultation was obtained and recorded. The primary outcome of this study was the rate of all-cause mortality, whilst incidence of HF rehospitalization was the secondary outcome. Rehospitalization for HF was defined as an unplanned readmission caused by decompensation of HF and presented as at least two of the following three manifestations: namely, decompensation of cardiac symptoms (dyspnea, rales, edema or elevated central venous pressure), NT-pro-BNP level ≥ 3 times the upper reference limit (URL) (URL = 100 pg/mL) and requiring treatment with intravenous diuretics [18].

### 3.5. Statistical Analysis

All statistical tests and confidence intervals (CI) were two-sided with *p* < 0.05 considered as statistically significant. Most of the statistical analyses were performed with SPSS software 19.0. Data were presented as the percentage or mean ± standard deviation (SD). Student’s *t*- or correction *t*-tests or one-way ANOVA was used where appropriate to compare means for continuous variables, while Chi-square analysis was used to compare the frequency for categorical variables. Univariate and multivariate logistic analysis and COX regression analysis were performed to identify the impact (or independent impact) of risk factors or different carnitines on HF diagnosis, DCM discrimination and prognosis prediction (multivariate analysis method: conditional backward). Receiver operating characteristic curve (ROC), one of most common methods to verify model discrimination, was analyzed to identify HF diagnosis or DCM prediction. Calibration plot and Hosmer-Lemeshow goodness-of-fit test were applied to verify model calibration. The statistical difference between two ROCs was analyzed with software Medcalc 19.0.7.

## 4. Discussion

Taking advantage of sensitive and precise mass spectrometry (MS) methods, this study quantified the carnitine profiles in control and HF patients consisting of both free carnitine (C0) and acylcarnitines, including short-chain, medium-chain and long-chain samples. In the present study, we confirmed that several acylcarnitines were increased significantly in HF patients and associated with poor long-term prognosis independently. More intriguingly, we verified the diagnostic capability of acylcarnitines to differentiate non-ischemic DCM from IHD. To the best of our knowledge, this has not been reported so far. We found that not only long-chain acylcarnitines derived from fatty acids but also short-chain ones derived from bran-chain amino acids played a favorable role on clinical diagnosis and outcomes prediction.

ATP production in health heart is mostly derived from mitochondrial oxidative metabolism. The relative contributions of fatty acids and carbohydrates to energy provision for the health heart are approximately 70% and 30%, respectively [19]. However, in order to meet its enormous energy consumption, the metabolic flexibility of the myocardium is extraordinarily dynamic with its capability to rapidly switch the pattern of fuel utilization in a diverse status of physiopathology [19]. A disturbance in the myocardial metabolism leads to the accumulation or loss of specific metabolites, which can be reflected within systemic circulation [7,20]. As one of essential factors in transporting long-chain fatty acids, carnitine level and its alteration directly influence the efficiency of the mitochondrial fatty acids’ β-oxidation. Conversely, an irreversible remodel of fatty acids metabolism in HF also disturbs carnitines’ synthesis. Therefore, it will be quite natural to measure circulating carnitines and find their relation to cardiac outcomes in HF.

Being consistent with previous findings [7,21,22,23], serum acylcarnitines in our study were elevated significantly in HF patients. It was reported that this elevation might be associated with the injury and permeability perturbation of cardiomyocyte membrance, hence an increased leakage of carnitines [23]. However, there were still two findings in our study that were different from previous reports. First, free carnitine (C0), which was decreased in previous reports (including concentrations in myocardium or plasma level) [24], was slightly increased in our HF patients (increased 5.15% in IHD-HF and 14.11% in DCM-HF group). These discrepant results might be confounded by diverse baseline characteristics (such as age or LVEF). Second, we found the independent impact of short-chain and medium-chain acylcarnitines on HF diagnosis (C2-C5, C6-C12) or prognosis prediction (C4DC, isoC5) apart from long-chain ones (C18:1, C18:2) reported previously [6]. This implies that both the catabolism of fatty acids and the branch-chain amino acids are attributable to HF diagnosis and differentiation of IHD-HF and DCM-HF since C4DC and isoC5 (amongst C2-C5) are derived from catabolic processes of valine, isoleucine and leucine, collectively or respectively [9,10].

There were statistically significant differences in the age of the patients enrolled in the various groups. A previous study showed that age had no impact on plasma carnitine concentrations [25]. We performed a Pearson correlation analysis between age and carnitine levels in the control group and found only three carnitines (C5DC, MC4 and C16) were statistically associated with age. They were all negatively correlated with age (C5DC: r = −0.367, *p* = 0.009; MC4: r = −0.284, *p* = 0.046; C16: r = −0.314, *p* = 0.027). However, these carnitines were all elevated in heart failure patients. We also compared serum carnitine levels in the control group between genders and found seven carnitines (isoC5, MC4, C16, C18:2, C18:1, C18 and C18:1OH) were higher in males than females. A previous study [25] also showed that males had significantly higher endogenous plasma L-carnitine and total carnitine concentrations than females. In our study, multivariate logistic analysis was performed and demonstrated that, when adjusted simultaneously by age, creatinine, male gender and hypertension, several carnitines (including isoC5, MC4, C16, C18:2, C18:1 and C18) verified HF diagnosis independently. So, we believe that age and gender had no decisive influence on our conclusions.

Several experimental studies found that a carnitine deficiency in the myocardium was associated with the deterioration of cardiac dysfunction [26,27]. The protective role of myocardial carnitines was supported not only by studies in patients with genetic carnitine deficiency that resulted in special cardiomyopathy [27,28] but also by the clinical benefit of carnitine supplementation in HF patients [4]. However, more and more studies in circulating carnitines present discrepant results with markedly raised carnitine levels in HF patients, which may seem in contrast with the decrease of myocardial carnitines [7,21]. The elevation of circulating carnitines may be caused by an increased leakage of carnitine through damaged cardiomyocyte membranes. It has also been demonstrated that accumulation of acylcarnitines may be toxic to several organs, including heart, skeletal muscle and liver [29]. Long-chain acylcarnitines might have arrhythmogenic effects by altering the sarcolemmal α-receptor function, leading to the intracellular Ca^2+^ disturbance [30].

So far, most studies had analyzed circulating acylcarnitines in non-HF and HF patients [7]. Few studies were designed to compare the circulating carnitine profile in non-ishemic and ischemic HF. As one of the most crucial cardiovascular diseases, IHD had been the leading cause of cardiac mortality [31]. IHD and non-ischemic DCM, with distinct long-term prognoses [11,12], underwent totally different pathophysiological processes and myocardial injury. Furthermore, treatment strategies and responses to certain drugs or devices were also quite different [32]. Although coronary angiography has been one of the most critical methods to diagnose IHD, some other possibilities must be taken into account. First, IHD-HF diagnosis needs to combine angiographic results and an individual history of MI. It will be quite controversial when one suffers moderate coronary stenosis lesion. Second, there were quite a lot of absolute and relative contraindications for immediate angiography, which will also need to be postponed in patients with acute decompensated HF and acute renal dysfunction. In order to clarify the diagnostic probability of circulating carnitines, the sensitivity of acylcarnitines to differentiate severe HF from moderate ones, or HF with preserved ejection fraction from those with reduced ejection fraction, were analyzed in previous studies [33,34]. Even more, Pierpont M found that the left ventricular carnitine in HF patients was higher than the level in the right ventricle, and both ventricles had higher acylcarnitines than those in the atria [35]. Therefore, it will be promising to employ some independent metabolites or establish a composite model (clinical factors added with certain carnitines), which might benefit early diagnosis of non-ischemic or ischemic HF etiology rapidly and accurately.

It was the first time that circulating acylcarnitines profiles between ischemic (IHD) and non-ischemic (DCM) were derived in HF patients directly. Because baseline characteristics were different slightly between these two groups, hierarchical analysis and multivariate logistic analysis were applied. Although patients in the IHD-HF group were older than those in the DCM-HF group (61 vs. 65 years), DCM patients suffered more severe cardiac dysfunction (32.8% vs. 37.9%). After subgrouping by age and LVEF, multiple carnitines in the DCM group were still higher than those in the IHD group, which indicated that a higher level of serum acylcarnitines in the non-ischemic DCM was not only contributed by the different ages and the extent of cardiac dysfunction. Forest plots results (Figure 3) and multivariate logistic analysis (Table 4) demonstrated that seven acylcarnitines, including short- (C2, C3, isoC4), medium- (C6) and long-chain (C18, C18:1, C18:2) acylcarnitines, significantly increased the diagnostic probability of DCM in these HF patients. ROC analysis indicated that the novel model comprised of isoC4, C18 and clinical factors (established by multivariate logistic regression analysis) achieved the AUC 0.832, which was higher than any carnitine or clinical model (Figure 4A). These results suggest that special acylcarnitines did increase the diagnostic efficiency of DCM from the total IHD and non-ischemic DCM HF patients.

## 5. Study Limitations

There were several limitations in the study. First, the sample number was relatively small, which might weaken the stability of our conclusion. Fortunately, even adjusted by multivariable analyses, quite a few acylcarnitines also achieved statistical significance. Second, 12 HF patients (6 in IHD-HF group and 6 in DCM-HF group) were loss to follow up. It will influence our results partially. Third, circulating carnitines were measured only at the baseline point. If dynamic detection were added at different time points after clinical therapy, these results would provide more and more causal evidence of carnitines in HF patients. These dynamic measurements will be taken into account in our future research.

## 6. Conclusions

Serum carnitines could not only serve as diagnostic and predictive biomarkers in HF but also benefit identification of HF primary etiology and prognosis.

## Figures and Tables

**Table 1:** Baseline Characteristics of the study.

	Control Group (*n* = 48)	IHD-HF Group (*n* = 63)	DCM Group (*n* = 98)
Age (years)	47 ± 16	65 ± 9 *	61 ± 14 *#
Male (%)	21 (43.8)	58 (92.1) *	83 (84.7) *
Heart rate (beats/min)	77 ± 11	77 ± 15	76 ± 14
Hypertension (%)	1 (2.1)	35 (55.6) *	40 (40.8) *
Diabetes (%)	1 (2.1)	18 (28.6) *	23 (23.5) *
History of PCI/CABG (%)	0 (0)	30 (47.6)	0 (0)
History of MI	0 (0)	26 (41.3)	0 (0)
Systolic BP (mmHg)	121 ± 13	127 ± 21	118 ± 25 #
Diastolic BP (mmHg)	77 ± 9	79 ± 12	76 ± 13
Blood glucose (mmol/L)	4.91 ± 0.58	6.95 ± 3.45 *	6.16 ± 2.31 *
HbA1c (%)	5.44 ± 0.41	6.45 ± 1.34 *	6.27 ± 0.99 *
D-dimer (mg/L)	0.27 ± 0.23	0.73 ± 1.19 *	0.80 ± 1.11 *
Hemoglobin (g/L)	135 ± 17	136 ± 18	137 ± 20
Serum creatinine (μmol/L)	69.3 ± 16.7	100.8 ± 29.7 *	107.2 ± 46.6 *
Serum Uric acid (μmol/L)	308.9 ± 77.9	437.7 ± 123.8*	480.1 ± 137.9 *#
Total cholesterol (mmol/L)	4.39 ± 0.94	3.67 ± 1.03 *	3.93 ± 1.04 *
Triglyceride (mmol/L)	1.39 ± 0.82	1.81 ± 1.31 *	1.56 ± 0.98
HDL-C (mmol/L)	1.22 ± 0.31	0.97 ± 0.22 *	1.11 ± 0.58
LDL-C (mmol/L)	2.56 ± 0.82	1.94 ± 0.82 *	2.22 ± 0.88 *#
NYHA (grade):			
I–II	48 (100.0)	54 (85.7) *	54 (55.1) *#
III–IV	0 (0)	9 (12.3) *	44 (44.9) *#
CRP (mg/L)	0.86 ± 1.01	8.89 ± 13.83 *	8.91 ± 16.10 *
Troponin T (ng/mL)	0.005 ± 0.006	0.093 ± 0.147 *	0.042 ± 0.041 *#
NT-pro-BNP (pg/mL)	66.5 ± 78.1	3402.4 ± 4170.6 *	4635.0 ± 5561.2 *
stenosis ≥ 75% & vessels ≥ 2	--	34 (54.0)	0 (0)
LM stenosis ≥ 75%	--	7 (11.1)	0 (0)
LAD stenosis ≥ 75%	--	26 (41.3)	0 (0)
Medication, *n* (%)			
ACEI/ARB	--	57 (90.5)	93 (94.9)
Beta-blocker	--	59 (93.7)	92 (93.9)
MRA	--	38 (60.3)	66 (67.3)
statins	--	58 (92.1)	23 (23.5) #

ACEI = angiotensin-converting enzyme inhibitor; ARB = angiotensin receptor blocker; BP = blood pressure; CABG = coronary artery bypass grafting; CRP = C-reactive protein; DCM = Dilated cardiomyopathy; HbA1c = glycosylated hemoglobin; HDL-C = high-density lipoprotein cholesterol; HF = Heart failure; IHD = Ischemia heart disease; LDL-C = low-density lipoprotein cholesterol; MRA = mineralocorticoid receptor antagonist; NT-pro-BNP = N-terminal pro–B-type natriuretic peptide; NYHA = New York Heart Association; PCI = percutaneous coronary intervention.* IHD-HF group vs. control group or DCM-HF group vs. control group, *p* < 0.05. # DCM-HF group vs. IHD-HF group, *p* < 0.05.

**Table 2:** The serum concentration of carnitines (nmol/L).

Carnitines	Abbreviation	Control Group (*n* = 48)	All HF Patients (*n* = 161)	IHD-HF Group (*n* = 63)	DCM-HF Group (*n* = 98)
L-Carnitine	C0	6468.6 ± 1294.1	7154.0 ± 1990.8 *	6799.9 ± 1891.6	7381.6 ± 2028.95 *
O-Acetyl-L-carnitine	C2	6157.3 ± 1920.3	10,595.1 ± 6154.2 *	9140.4 ± 4862.7 *	11530.3 ± 6715.7 *#
Propionyl-L-carnitine	C3	437.6 ± 218.8	698.0 ± 450.8 *	564.1 ± 205.3	783.0 ± 537.7 *#
Butyryl-L-carnitine	C4	114.4 ± 43.6	184.8 ± 98.1 *	163.7 ± 84.8 *	198.4 ± 103.9 *#
Isobutyryl-L-carnitine	isoC4	142.9 ± 110.3	314.5 ± 252.2 *	252.5 ± 177.1 *	354.4 ± 284.2 *#
Isovaleryl-L-carnitine	isoC5	106.3 ± 35.5	142.4 ± 64.1 *	131.9 ± 58.5 *	149.2 ± 66.8 *
2-Methylbutyryl-L-Carnitine	MC4	43.1 ± 17.8	76.2 ± 47.2 *	69.0 ± 37.5 *	80.9 ± 52.5 *
Methylmalonyl DL-Carnitine	MM	b.LOD	b.LOD	b.LOD	b.LOD
Valeryl-L-carnitine	C5	2.03 ± 2.06	4.56 ± 4.08 *	3.75 ± 2.35 *	5.08 ± 4.82 *#
3-Hydroxybutyrylcarnitine	C4OH	28.8 ± 21.4	100.1 ± 117.2 *	78.5 ± 101.0 *	114.0 ± 125.0 *
Hexanoyl-L-carnitine	C6	49.2 ± 29.5	105.7 ± 75.5 *	86.6 ± 63.4 *	117.9 ± 80.4 *#
Succinyl Carnitine	C4DC	20.8 ± 7.6	46.2 ± 23.5 *	42.0 ± 22.7 *	49.0 ± 23.7 *
(2R)-3-Hydroxyisovaleroyl Carnitine	C5OH	18.1 ± 5.1	35.4 ± 30.1 *	30.2 ± 17.5 *	38.7 ± 35.6 *
L-Glutaryl Carnitine	C5DC	316.0 ± 141.6	503.9 ± 397.4 *	492.1 ± 346.4 *	511.4 ± 428.6 *
Octanoyl-L-carnitine	C8	97.7 ± 85.3	159.8 ± 101.3 *	147.1 ± 100.7 *	168.0 ± 101.3 *
Adipoyl-L-carnitine	C6DC	12.6 ± 8.2	41.5 ± 52.7 *	35.5 ± 43.5 *	45.3 ± 57.8 *
Decanoyl-L-carnitine	C10	127.9 ± 136.1	198.6 ± 131.5 *	184.8 ± 139.2 *	207.5 ± 126.2 *
Lauroyl-L-carnitine	C12	18.4 ± 16.9	37.0 ± 32.4 *	35.1 ± 37.2 *	38.2 ± 29.0 *
5-cis-Tetradecenoyl Carnitine	C14:1	41.5 ± 30.1	98.5 ± 105.8 *	88.6 ± 122.2 *	104.9 ± 94.0 *
Myristoyl-L-carnitine	C14	1.75 ± 4.33	11.88 ± 15.38 *	10.00 ± 17.8 *	13.1 ± 13.6 *
Palmitoyl-L-carnitine	C16	95.8 ± 24.1	146.2 ± 55.62 *	129.7 ± 45.0 *	156.8 ± 59.3 *#
3-Hydroxyhexadecanoyl-Carnitine	C16OH	b.LOD	b.LOD	b.LOD	b.LOD
Linoleoyl-L-carnitine	C18:2	169.0 ± 54.8	332.4 ± 156.3 *	278.3 ± 105.3 *	367.2 ± 173.4 *#
Oleoyl L-carnitine	C18:1	154.1 ± 44.9	297.5 ± 140.9 *	246.2 ± 93.3 *	330.6 ± 156.1 *#
Stearoyl-L-carnitine	C18	45.5 ± 9.0	63.0 ± 22.9 *	54.5 ± 16.9 *	68.5 ± 24.5 *#
3-Hydroxyoleylcarnitine	C18:1OH	1.12 ± 0.85	2.43 ± 2.13 *	2.10 ± 1.90 *	2.64 ± 2.25 *
Arachidonoyl-L-carnitine	C20:4	32.1 ± 14.2	74.0 ± 46.2 *	64.0 ± 31.8 *	80.4 ± 52.7 *#

DCM = Dilated cardiomyopathy; HF = Heart failure; IHD = Ischemia heart disease; b.LOD = below detection limit. * IHD-HF group vs. control group or DCM-HF group vs. control group, *p* < 0.05. **#** DCM-HF group vs. IHD-HF group, *p* < 0.05.

**Table 3:** Multi-variable logistic analysis of a series of carnitines for the risk of heart failure (LVEF < 55%).

	ROC		Unadjusted			Adjusted by ACGH		
	C Value	*p* Value	OR	95% CI	*p* Value	OR	95% CI	*p* Value
NT-pro-BNP	0.995	<0.01	1.019	1.010–1.028	<0.01	1.020	1.008–1.033	<0.01
C0	0.614	0.017	1.000	1.000–1.000	0.027	1.000	1.000–1.001	<0.01
C2	0.794	<0.01	1.000	1.000–1.001	<0.01	1.000	1.000–1.001	<0.01
C3	0.776	<0.01	1.005	1.003–1.007	<0.01	1.004	1.001–1.007	<0.01
C4	0.769	<0.01	1.021	1.012–1.031	<0.01	1.021	1.008–1.033	<0.01
isoC4	0.805	<0.01	1.009	1.005–1.014	<0.01	1.006	1.002–1.010	<0.01
isoC5	0.670	<0.01	1.013	1.006–1.021	<0.01	1.011	1.001–1.021	0.031
MC4	0.806	<0.01	1.059	1.034–1.085	<0.01	1.038	1.008–1.068	0.012
C5	0.768	<0.01	1.575	1.290–1.294	<0.01	1.455	1.090–1.944	0.011
C4OH	0.820	<0.01	1.048	1.026–1.071	<0.01	1.041	1.011–1.071	<0.01
C6	0.829	<0.01	1.042	1.025–1.059	<0.01	1.032	1.013–1.052	<0.01
C4DC	0.915	<0.01	1.224	1.145–1.308	<0.01	1.211	1.105–1.328	<0.01
C5OH	0.835	<0.01	1.218	1.133–1.311	<0.01	1.236	1.115–1.369	<0.01
C5DC	0.695	<0.01	1.004	1.002–1.007	<0.01	--	--	NS
C8	0.762	<0.01	1.012	1.005–1.018	<0.01	--	--	NS
C6DC	0.848	<0.01	1.149	1.088–1.213	<0.01	1.121	1.045–1.203	<0.01
C10	0.746	<0.01	1.007	1.002–1.011	<0.01	--	--	NS
C12	0.766	<0.01	1.059	1.029–1.090	<0.01	--	--	NS
C14:1	0.785	<0.01	1.032	1.017–1.047	<0.01	1.018	1.002–1.034	0.027
C14	0.763	<0.01	1.189	1.108–1.276	<0.01	1.155	1.051–1.269	<0.01
C16	0.812	<0.01	1.040	1.025–1.055	<0.01	1.045	1.021–1.069	<0.01
C18:2	0.868	<0.01	1.020	1.013–1.027	<0.01	1.020	1.010–1.030	<0.01
C18:1	0.870	<0.01	1.023	1.015–1.032	<0.01	1.022	1.011–1.033	<0.01
C18	0.753	<0.01	1.073	1.041–1.105	<0.01	1.084	1.029–1.142	<0.01
C18:1OH	0.742	<0.01	2.329	1.596–3.397	<0.01	--	--	NS
C20:4	0.871	<0.01	1.087	1.057–1.118	<0.01	1.079	1.039–1.121	<0.01

ACGH = Age, Creatinine, Gender and Hypertension; CI: confidence interval; LVEF = left ventricular ejection fraction. NS: Non-significant Statistic difference (*p* > 0.05); NT-pro-BNP = N-terminal pro–B-type natriuretic peptide; OR: odds ratio.

**Table 4:** ROC analysis and Logistic analysis for DCM diagnosis.

	ROC		Unadjusted			Adjusted by ALU			Adjusted by ALU and LVEF		
	C Value	*p* Value	OR	95% CI	*p* Value	OR	95% CI	*p* Value	OR	95% CI	*p* Value
BNP	0.587	0.064	--	--	NS	--	--	NS	--	--	NS
Troponin T	0.478	0.645	0.002	0.000–0.209	<0.01	0.001	0.000–0.136	<0.01	0.000	0.000–0091	<0.01
C2	0.627	<0.01	1.000	1.000–1.000	0.021	1.000	1.000–1.000	0.020	1.000	1.000–1.000	0.039
C3	0.642	<0.01	1.002	1.001–1.003	<0.01	1.002	1.001–1.003	<0.01	1.002	1.001–1.004	<0.01
C4	0.615	0.014	1.004	1.000–1.008	0.034	--	--	NS	--	--	NS
isoC4	0.610	0.019	1.002	1.000–1.004	0.016	1.004	1.001–1.006	<0.01	1.004	1.001–1.007	<0.01
C5	0.565	0.163	1.116	1.000–1.246	0.050	--	--	NS	--	--	NS
C6	0.625	<0.01	1.006	1.001–1.013	0.014	1.010	1.002–1.018	0.011	1.009	1.001–1.017	0.019
C16	0.642	<0.01	1.011	1.003–1.018	<0.01	1.010	1.002–1.019	0.016	--	--	NS
C18:2	0.645	<0.01	1.004	1.002–1.007	<0.01	1.005	1.002–1.008	<0.01	1.004	1.001–1.007	0.010
C18:1	0.668	<0.01	1.006	1.002–1.009	<0.01	1.006	1.002–1.010	<0.01	1.005	1.001–1.009	<0.01
C18	0.676	<0.01	1.034	1.015–1.053	<0.01	1.032	1.011–1.053	<0.01	1.028	1.007–1.050	<0.01
C20:4	0.560	0.200	1.009	1.001–1.017	0.033	--	--	NS	--	--	NS

ALU = Age, LDL-C and Uric acid; CI: confidence interval; LVEF = left ventricular ejection fraction; NS: Non-significant Statistic difference (*p* > 0.05); OR: odds ratio.

**Table 5:** Univariate and multivariate COX regression for independent risk factors analysis.

Univariate COX Regression	For Mortality		For HF Rehospitalization	
	HR (95% CI)	*p* Value	HR (95% CI)	*p* Value
age > 65	1.978 (1.056–3.704)	0.033	1.793 (1.037–3.099)	0.037
serum creatinine > 2 mg/dL	7.687 (3.191–18.518)	<0.01	3.572 (1.415–9.016)	<0.01
NT-pro-BNP > 4000 pg/mL	3.184 (1.705–5.947)	<0.01	2.659 (1.556–4.543)	<0.01
left atrial diameter > 55 mm	2.847 (1.482–5.467)	<0.01	2.410 (1.325–4.382)	<0.01
LVEF < 35%	1.106 (0.607–2.014)	0.742	3.380 (1.836–6.221)	<0.01
diagnosis of DCM	0.822 (0.449–1.507)	0.527	1.900 (1.033–3.495)	0.039
**Multivariate** **COX regression**	Unadjusted		Adjusted	
	HR (95% CI)	*p* value	HR (95% CI)	*p* value
**For all-cause mortality ^†^**				
C18:1 > 300 nmol/L	2.402 (1.309–4.406)	<0.01	2.363 (1.122–4.976)	0.024
isoC5 < 100 nmol/L	2.483 (1.353–4.556)	<0.01	2.108 (1.091–4.074)	0.026
**For HF rehospitalization ^‡^**				
C18:2 > 420 nmol/L	2.783 (1.607–4.822)	<0.01	2.088 (1.071–4.067)	0.031
C4DC > 60 nmol/L	2.964 (1.691–5.193)	<0.01	2.121 (1.002–4.490)	0.049
isoC5 < 100 nmol/L	2.277 (1.308–3.964)	<0.01	2.302 (1.282–4.132)	0.010

†: adjusted by age > 65, serum creatinine > 2 mg/dL, NT-pro-BNP > 4000 pg/mL and left atrial diameter > 55 mm; ‡: adjusted by age > 65, diagnosis of DCM-HF, LVEF < 35%, left atrial diameter > 55 mm, serum creatinine > 2 mg/dL and NT-pro-BNP > 4000 pg/mL.

## Data Availability

The data presented in this study are available on request from the corresponding author. The data are not publicly available due to privacy.
